# Development of global health research in China

**DOI:** 10.7189/jogh.07.020102

**Published:** 2017-12

**Authors:** Yan Guo

**Affiliations:** School of Public Health, Peking University, Beijing, China

With the continuous deepening and broadening of China’s engagement in global health as well as the transformation of its role in global health governance, global health science have made great strides in China, from the infancy stage of last century to the grown-up stage of this century. Considerable progress in global health discipline has been witnessed, especially in the last ten years. The rapid development of global health in China is characterized with three crucial indications: the rise of global health institutions, the ever-expanding research team and the growing number of global health researchers, and also the increasing number of relevant papers published in international journals.

Ten years ago, there was not any domestic institution specializing in global health research, let alone a nationwide research network in global health. In 2007, Peking University took the lead in establishing the Global Health Research Center, which became China's earliest research institute in this field. Subsequently, other universities, such as Fudan University, Wuhan University, Duke Kunshan University, Zhejiang University, Central South University, Sun Yat-sen University and so on, set up global health centers or research institutes. In 2011, the undergraduate major in global health was set up in Wuhan University. In 2012, the first global health department nationwide came into being under the School of Public Health, Peking University. In addition to those universities, China National Health Development Research Center and Chinese Center for Disease Control and Prevention (China CDC), as the official policy research institute and national public health agency under the leadership of National Health and Family Planning Commission of China (NHFPC), have also set up divisions that engage in global health research or practice. On this basis, ten universities jointly initiated the Chinese Consortium of Universities for Global Health (CCUGH) in 2013. It is the first cross-school organization in global health research field in China. Soon afterwards, in 2015, the China Global Health Network was established, involving not only universities but also government agencies, China CDC, pharmaceutical companies and other research and development (R&D) organizations. In 2016, the Global Health Branch was set up under Chinese Preventive Medicine Association. With the support of NHFPC, the Ministry of Commerce, and some international organizations such as China Medical Board (CMB) and UK Department for International Development (DFID), Chinese academia sets off an upsurge of research into global health, systematically studying how China's experience and lessons learnt in health development can be shared with other developing countries, exploring global health governance and health development assistance. Additionally, Fudan University and China CDC have also started pilot projects on maternal and child health and malaria control in Myanmar and Ethiopia. Based on all those researches, more and more articles on global health are being published.

The development of global health research in China should be attributed to three major contributing factors.

First, under the context of China’s overall development and diplomatic development, its practice in global health over the years play a key role. As early as 1963, China started to dispatch overseas medical teams to Algeria. Chinese medical teams are now working in 49 countries. The health cooperation between China and other countries has also extended to the public health field since 2013. For example, health professionals were dispatched to combat Ebola and Middle East respiratory syndrome (MERS) coronavirus and went to Madagascar to fight against plague. China also cooperated with the US CDC to help African countries set up their own CDCs. All of those practical efforts lay a good foundation for Chinese academia to carry out global health researches.

Second, investments made by the Chinese government in scientific research and the scientific research ability of Chinese researchers are improving. In 2016, a total of about yuan 1.57 trillion was invested in R&D, an increase of yuan 150.69 billion or 10.6% over the previous year [1]. In 2013, the expenditure on health research and development in China reached 46.84 billion yuan, accounting for 4.11% of the total nationwide R&D expenditure [2]. The increasing research expenditure has created a material foundation for Chinese researchers.

Third, extensive international cooperation in line with the reform and opening-up policy is a prerequisite for the development of global health research in China. With the deepening of reform and opening-up, the exchanges between China and the world are getting more and more frequent. As General Secretary Xi Jinping noted in the report to the 19^th^ CPC National Congress, the major-country diplomacy with distinctive Chinese features should promote the building of a new type of international relation and the fostering of a community of shared future for mankind [3]. An increasing number of Chinese scholars are joining in international cooperation. These exchange and cooperation processes also provide opportunities for global health researchers.

Despite progress made so far, China is still facing challenges in global health research. It is manifested from several aspects. Global health is still a relatively new concept in China. As an interdisciplinary field, it requires attention from various disciplines. However, current researchers in this field are mainly public health professionals and the capacity of personnel engaged in global health research needs to be enhanced. Moreover, it is necessary for more people to go board and identify needs for this field so that China can cooperate with more international counterparts and provide public health products worldwide. Therefore, there is still some way to go for China in global health. Nevertheless, as China marches on in reform and opening-up and actively takes more and more international responsibilities, it is believed that more people will join in the global health field, facilitate global health research and present more research achievements to the international community.
